# Repeatability of Radiomic Features in Non-Small-Cell Lung Cancer [^18^F]FDG-PET/CT Studies: Impact of Reconstruction and Delineation

**DOI:** 10.1007/s11307-016-0940-2

**Published:** 2016-02-26

**Authors:** Floris H. P. van Velden, Gerbrand M. Kramer, Virginie Frings, Ida A. Nissen, Emma R. Mulder, Adrianus J. de Langen, Otto S. Hoekstra, Egbert F. Smit, Ronald Boellaard

**Affiliations:** 1 Department of Radiology and Nuclear Medicine, VU University Medical Center, PO Box 7057, 1007MB Amsterdam, The Netherlands; 2 Department of Radiology, Leiden University Medical Center, Leiden, The Netherlands; 3 Department of Pulmonary Diseases, VU University Medical Center, Amsterdam, The Netherlands; 4 Department of Thoracic Oncology, Netherlands Cancer Institute, Amsterdam, The Netherlands; 5 Department of Nuclear Medicine and Molecular Imaging, University Medical Center Groningen, Groningen, The Netherlands

**Keywords:** PET/CT, Repeatability, Radiomics, Tracer uptake heterogeneity, Non-small-cell lung cancer (NSCLC)

## Abstract

**Purpose:**

To assess (1) the repeatability and (2) the impact of reconstruction methods and delineation on the repeatability of 105 radiomic features in non-small-cell lung cancer (NSCLC) 2-deoxy-2-[^18^F]fluoro-D-glucose ([^18^F]FDG) positron emission tomorgraphy/computed tomography (PET/CT) studies.

**Procedures:**

Eleven NSCLC patients received two baseline whole-body PET/CT scans. Each scan was reconstructed twice, once using the point spread function (PSF) and once complying with the European Association for Nuclear Medicine (EANM) guidelines for tumor PET imaging. Volumes of interest (*n* = 19) were delineated twice, once on PET and once on CT images.

**Results:**

Sixty-three features showed an intraclass correlation coefficient ≥ 0.90 independent of delineation or reconstruction. More features were sensitive to a change in delineation than to a change in reconstruction (25 and 3 features, respectively).

**Conclusions:**

The majority of features in NSCLC [^18^F]FDG-PET/CT studies show a high level of repeatability that is similar or better compared to simple standardized uptake value measures.

**Electronic supplementary material:**

The online version of this article (doi:10.1007/s11307-016-0940-2) contains supplementary material, which is available to authorized users.

## Introduction

Lung cancer is the leading cause of cancer death, with more than 1.6 million deaths worldwide in 2012 [1]. About 80–85 % of the cases are classified as non-small-cell lung cancer (NSCLC) [2]. Early assessment of response to treatment (e.g., radiotherapy and/or chemotherapy) is essential to determine which patients will benefit from treatment and which may require treatment adaptations, paving the way for personalized cancer therapies [3]. Several studies have demonstrated the potential of positron emission tomography/computed tomography (PET/CT) to assess the effects of treatment for NSCLC patients early using 2-deoxy-2-[^18^F]fluoro-D-glucose ([^18^F]FDG) [4–7]. Although the benefits of new response metrics such as the metabolically active tumor volume (MATV) and total lesion glycolysis (TLG) are currently under investigation, response to treatment is predominantly measured using the maximum standardized uptake value (SUV_max_) obtained within a tumor [8]. However, SUV_max_ is not capable to capture all forms of responses accurately. For instance, SUV_max_ can only measure response accurately if there is a global change in tracer uptake, i.e., in absence of a spatially heterogeneous response [9]. In addition, since SUV_max_ only involves a single voxel, it is inherently unable to capture intratumor heterogeneity and unable to measure a change in the shape or volume of (the metabolically active part of) the tumor. In recent years, various advanced quantitative imaging features, so-called radiomic features, have been proposed and investigated for their potential to quantify tracer uptake, tracer uptake heterogeneity, and/or (metabolically active) tumor geometry [9–21]. The term radiomics refers to studies that extract a large amount of advanced quantitative imaging features from medical imaging studies, e.g., PET/CT studies, as a basis for characterizing a specific aspect of patient health [22–24].

Several challenges have been identified that need to be addressed before radiomic features can be used in clinical practice, including the standardization and robustness of selected features [21]. For standardization, it is of utmost importance to identify which radiomic features are sensitive to a change in reconstruction settings [25–27] or to a change in delineation [11, 26]. For instance, radiomic features that can characterize tracer uptake heterogeneity may treat both partial volume effects and noise as heterogeneity [9]. Although it has been shown that several radiomic features are not sensitive to partial volume effects and noise when extracted from PET/CT response data of esophageal carcinoma patients [26], it has been shown that some features do require image denoising and partial volume correction prior to extraction [9, 26]. Recently, two studies [25, 27] investigated the effects of different reconstruction settings on the values obtained from various texture-based features and indicated a need for standardization. Note that for response monitoring studies, it is important to know whether an observed change in tracer uptake, tumor geometry, or tracer uptake heterogeneity is due to a true response or methodological variation (i.e., biological, technical, or observer variability). Therefore, it is essential to assess the repeatability of these radiomic features. However, to the best of our knowledge, the effects of reconstruction and delineation on the repeatability of a large set of radiomic features, including intensity-, shape-, and texture-based features, have not yet been assessed.

Therefore, the aim of this study was to assess the repeatability of various radiomic features in NSCLC [^18^F]FDG-PET/CT studies, taking different reconstruction settings and delineation methods into account. To assess the impact of different reconstruction settings, PET data were reconstructed twice using settings that either ensure harmonization (i.e., complied with the European Association of Nuclear Medicine (EANM) guidelines for tumor PET imaging [28]) or are more state of the art (i.e., use of a resolution model during image reconstruction). To assess the impact of delineation, volumes of interest (VOIs) were defined manually on (low-dose) CT images and semi-automatically on PET images. CT-based delineation was explored to illustrate the effects when using the anatomical volume of a tumor, thereby potentially capturing a higher level of tracer uptake heterogeneity within a VOI (e.g., by the inclusion of necrotic areas) compared to semi-automatic threshold-based isocontour methods on PET data.

## Materials and Methods

### Patient Data

Eleven NSCLC patients (Table 1) received double-baseline whole-body [^18^F]FDG scans that were acquired on a time-of-flight (TOF) PET/CT scanner (Philips Healthcare, Cleveland, OH). The time interval between first and second baseline scans was less than 3 days (1.3 ± 0.5 days). This prospective study has been approved by the institutional review board and is part of a study that has been registered in the Dutch trial register (www.trialregister.nl; NTR3508). Informed consent was obtained from all individual participants included in the study. Patients were included if they were 18 years or older, were diagnosed with stage IIIB or IV of NSCLC, had at least one lesion with a diameter larger than 3 cm, and were able to remain supine for 60 min during acquisition. Patients were excluded if they were pregnant or lactating, had chemotherapy in the past 4 weeks, metal implants, a body weight of more than 100 kg, or known diabetes mellitus type I or II.

**Table 1:** Patient demographics

Parameter	Value
Gender	
Male	7
Female	4
Age (year)	
Median	61
Range	45–66
Weight (kg)	
Median	76
Range	57–114
Stage	
III B	4
IV	7
Histology	
Adenocarcinoma	8
Squamous cell carcinoma	3
Type of lesion	
Primary	6
Metastasis	13
Localization	
Lung	5
Mediastinum	8
Hilum	2
Clavicular region	4
Lesion volume (ml)	
Median	39
Range	18–702

### Acquisition, Reconstruction, and Post-Processing

A static whole-body emission scan was started 1 h (61 ± 2 min) after injection of [^18^F]FDG (263 ± 61 MBq). Prior to this emission scan, a low-dose CT scan (120 kVp, 50 mAs) was acquired during normal breathing. All PET data were normalized and corrected for scatter and random events, dead time, attenuation, and decay and reconstructed twice using vendor-recommended reconstruction settings. All reconstruction settings utilize a blob-based TOF list-mode-ordered subset expectation maximization algorithm with 3 iterations and 33 subsets [29]. The first reconstruction setting applied an additional Gaussian filter in order to comply with the EANM guidelines for tumor PET imaging [28]. The second reconstruction setting applied an additional post-reconstruction resolution recovery method, i.e., a maximum likelihood expectation maximization deconvolution [30] that uses the spatially variant point spread function (PSF) of the system, as implemented by the PET/CT vendor. All resulting PET images have a matrix size of 144 × 144 voxels with a voxel size of 4 × 4 × 4 mm. After reconstruction, PET image data were expressed in SUV by normalizing voxel radioactivity concentrations [kBq · ml^−1^] to injected dose of [^18^F]FDG [MBq] and the patient’s body weight (kg). All CT images have a matrix size of 512 × 512 voxels with a voxel size of 1.2 × 1.2 × 5 mm and were rescaled to the dimensions of the PET images prior to delineation. In this way, voxel tissue fraction effects within the delineations are avoided and calculations are performed using the original non-rebinned PET images, as recommended by Uniform Protocols for Imaging in Clinical Trials (UPICT) working group [31].

### Delineation

Nineteen VOIs were delineated for lesions larger than 10 ml on both PET and low-dose CT images. CT-based VOIs were drawn manually upon consensus between an experienced physician, a physician in training, and a medical physic expert, using the medical history and previously acquired contrast-enhanced CT images as prior knowledge. PET-based VOIs were drawn semi-automatically by using an isocontour method that applies a threshold of 50 % of the 3D peak SUV (SUV_peak_, obtained using a sphere of 12-mm diameter) corrected for local background [12]. PET-based VOIs were drawn twice, i.e., both on PSF-based and EANM-compliant images.

### Radiomic Features

For each VOI, 105 radiomic features were determined. These features can be divided into the following three groups (Table 2): intensity (*n* = 27), shape (*n* = 9), and texture (*n* = 69). The textural features were based on fractals, grey-level co-occurrence matrices (GLCMs), or grey-level run-length matrices (GLRMs). Features derived from GLCM and GLRM were calculated by averaging the obtained values over 13 symmetric directions in three dimensions [11]. For those features that require SUV discretization (i.e., resampling of the image intensity values), two types of discretization were used [21], 64 grey-level bins [14, 18] or a fixed bin size of 0.25 g/ml [21]. A fixed bin size of 0.25 g/ml represents the mean SUV_max_ for all 19 lesions (18 and 14 g/ml when obtained from PSF-based and EANM-compliant images, respectively) divided by 64 bins.

**Table 2:** Implemented radiomic features with corresponding literature references describing the features

Group	No. of features	Names of radiomic features	Described in
Intensity	27	Maximum standardized uptake value (SUV_max_), mean SUV (SUV_mean_), mean SUV of a sphere of 12-mm diameter (SUV_peak_), coefficient of variation (COV), total lesion glycolysis (TLG), mean SUV of maximum SUV and the six adjacent voxels (SUV_star_), minimum SUV (SUV_min_), range of SUV (SUV_range_), median SUV (SUV_median_), standard deviation (SD), skewness, kurtosis, mean absolute deviation, median absolute deviation, mean Laplacian, total energy, variance, root-mean-square (RMS), Moran’s I, Geary’s C, uniformity^a^, entropy^a^, local entropy^a^, and area under a cumulative (AUC) SUV-volume histogram	[9–14]
Shape	9	Compactness A, compactness B, sphericity, disproportion, surface area, metabolically active tumor volume (MATV) or anatomical volume (AV), surface to volume ratio (S2V), surface of an equivolumetric sphere to volume ratio (S2V_eq_), and radius of an equivolumetric sphere	[11]
Texture	69	Based on fractals (*n* = 3): fractal dimension (FD), abundance, and lacunarity; Based on grey-level co-occurrence matrices^a^ (*n* = 44): autocorrelation, cluster prominence, cluster shade, cluster tendency, contrast, correlation, difference entropy, dissimilarity, energy, entropy, homogeneity 1, homogeneity 2, informational measure of correlation 1 (IMC1), IMC2, inverse difference moment normalized (IDMN), inverse difference normalized (IDN), inverse variance, maximum probability, sum average, sum entropy, sum variance, and variance; Based on grey-level run-length matrices^a^ (*n* = 22): grey-level non-uniformity (GLN), high-grey-level run emphasis (HGLRE), long-run emphasis (LRE), long-run high-grey-level emphasis (LRHGLE), long-run low-grey-level emphasis (LRLGLE), low-grey-level run emphasis (LGLRE), run length non-uniformity (RLN), run percentage (RP), short-run emphasis (SRE), short-run high-grey-level emphasis (SRHGLE), and short-run low-grey-level emphasis (SRLGLE)	[11, 15, 16]

^a^Two types of SUV discretization were used, 64 grey-level bins or a fixed bin size of 0.25 g/ml

### Statistics

To assess the level of repeatability, mean relative test-retest variability (TRT_*r*_, in %) was calculated for all 105 radiomic features by Eq. (1).

1 $$ {\mathrm{TRT}}_r=\frac{1}{n}\times {\displaystyle {\sum}_{i=1}^n\frac{\mathrm{test}\hbox{-} \mathrm{retest}}{\mathrm{mean}\left(\mathrm{test},\kern0.5em \mathrm{retest}\right)}\times 100\%} $$

where *n* is the number of lesions. In addition, mean absolute TRT (TRT_*α*_, in %) was calculated by TRT_*a*_ = |TRT_*r*_|. A TRT closer to zero indicates a higher level of repeatability. In addition, intraclass correlation coefficients (ICCs) were calculated between the values obtained from first and second baseline scans using a one-way random single-measure model (Real Statistics Resource Pack release 3.5; www.real-statistics.com). ICC does not only take the variance within subjects but also variance between subjects into account. An ICC of 1 indicates perfect reliability. For both TRT_*r*_ and ICC, 95 % confidence intervals were calculated. A related-sample Wilcoxon signed-rank test was applied to ICC, TRT_*r*_, and TRT_*a*_ of all features to assess whether a change in reconstruction setting or delineation significantly changed ICC, TRT_*r*_, or TRT_*a*_. *P* values less than 0.05 were considered significant.

A threshold of 0.90 for ICC was used to group features into sets of features showing an overall good, variable, or overall poor performance. This threshold is in line with the ICC found in literature for SUV_max_ [11, 13, 14]. An overall good performance means that all four possible combinations of delineation and reconstruction algorithm resulted in an ICC ≥ 0.90, whereas a variable performance means that at least one but not all combinations resulted in an ICC ≥ 0.90. An overall poor performance indicates that all combinations resulted in an ICC < 0.90. Features were considered to be sensitive to an applied delineation and/or selected reconstruction algorithm when the absolute change in ICC was at least 0.03. For these features, the best performing delineation and/or reconstruction algorithm was determined.

## Results

**Fig. 1:**
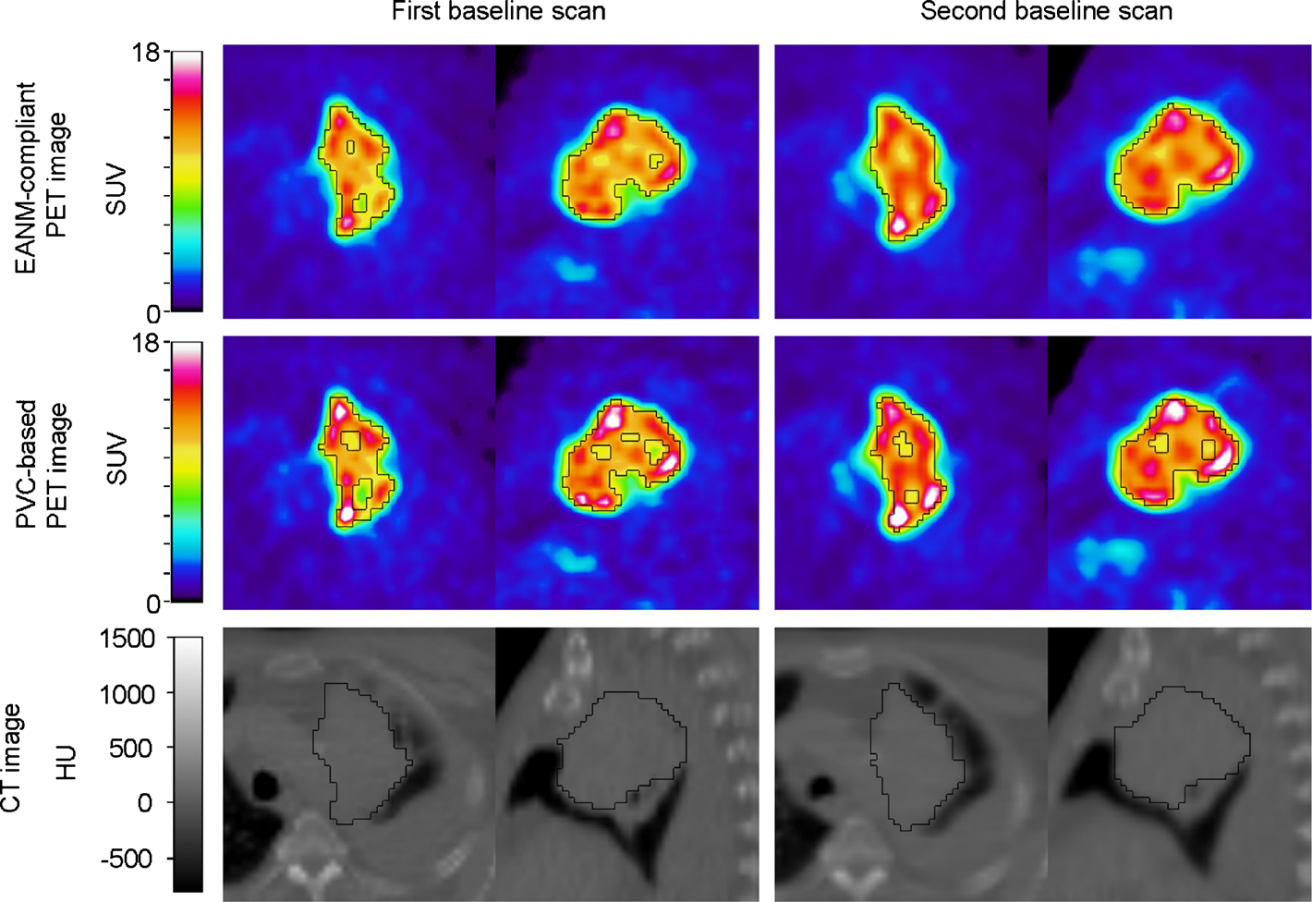
Axial (*left*) and sagittal (*right*) PET/CT images of a typical NSCLC patient with (visually) rather heterogeneous [^18^F] FDG uptake in the primary lung tumour. The *black contours* illustrate the various (CT- or PET-based) delineations. Rigid co-registration was applied for illustration purposes only to co-register the second baseline scan onto the first baseline scan using VINCI v4.23 (Max-Planck-Institute for Neurological Research, Cologne, Germany) (Color figure online).

**Fig. 2:**
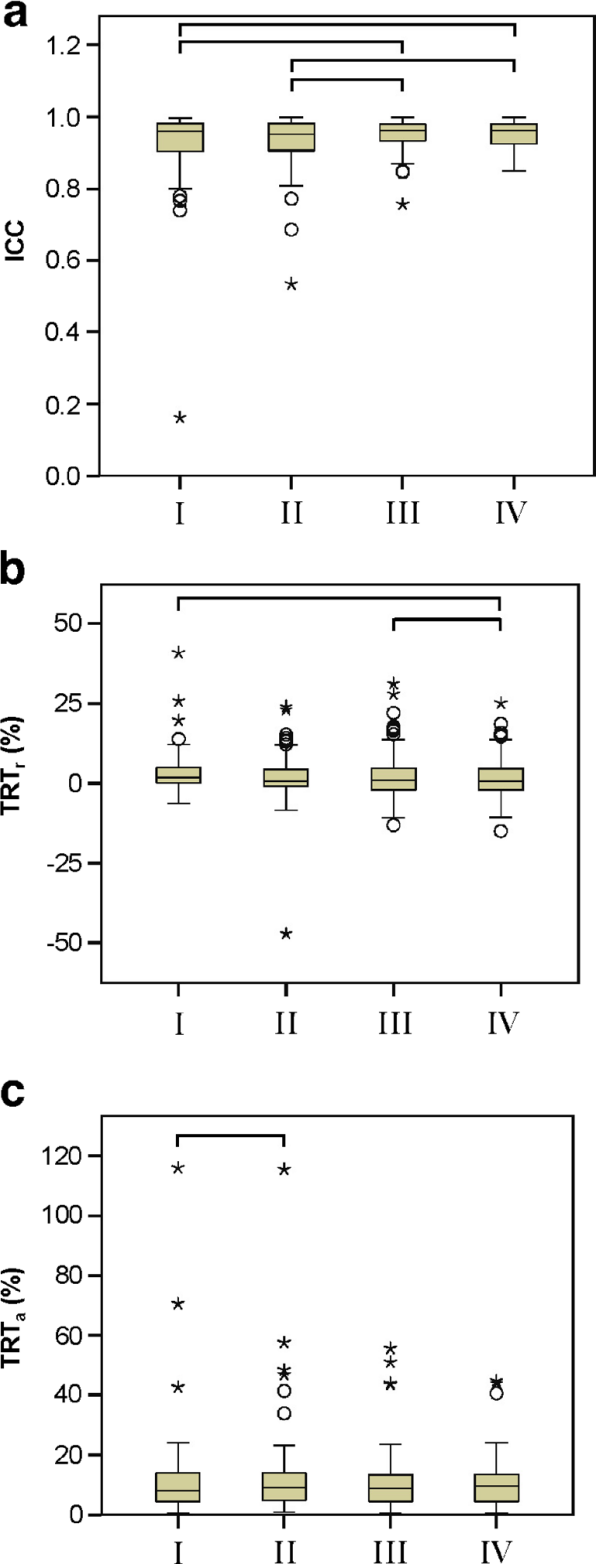
Box plots of **a** ICC, **b** TRT, and **c** TRT of radiomic features extracted from EANM-compliant reconstruction with (I) PET-based or (III) CT-based delineation or PSF-based reconstruction using (II) PET-based or (IV) CT-based delineation. *Circles* illustrate outliers, and *stars* illustrate extreme cases. A *bar* indicates a statistically significant difference (*p* value < 0.05).

Most intensity-, shape-, and texture-based features (98 %) have a repeatability that is comparable to those seen for simple SUV measures in literature (e.g., SUV_max_, SUV_mean_, and SUV_peak_) (Supplemental Tables 1 to 12). When compared to the ICC of SUV_mean_ observed in this study, 37 % of the features showed an equal or better ICC for at least one combination of delineation and reconstruction, while 12 % of the features showed an equal or better ICC independent of delineation and reconstruction. Figure 1 shows a typical example where the various reconstruction settings and image types (e.g., functional or anatomical) resulted in different VOI. A small but significant improvement in median ICC was observed for features extracted using CT-based delineation compared to those extracted using PET-based delineation independent of the applied reconstruction setting (from 0.960 to 0.962 and from 0.953 to 0.962 for EANM-compliant and PSF-based images, respectively; Fig. 2). This is also reflected in a decrease in the number of outliers and extreme cases (Table 3), derived from the box plots (Fig. 2). In addition, a small but significant improvement in median ICC was observed for features extracted using EANM-compliant reconstruction with CT-based delineation compared to those extracted using PSF-based reconstruction with PET-based delineation (from 0.953 to 0.962). All other differences in median ICC were insignificant.

**Table 3:** Outliers and extreme cases of radiomic features extracted from EANM-compliant or PSF-based reconstructed PET images using PET-based or CT-based delineation

Delineation	Reconstruction	ICC		TRT_*r*_		TRT_*a*_	
		Outliers	Extreme cases	Outliers	Extreme cases	Outliers	Extreme cases
PET-based	EANM-compliant	AUC	FD and homogeneities 1 and 2 (64B)	Variance	Cluster shade (64B and FB) and cluster prominence (64B)	–	Cluster prominence (64B and FB) and cluster shade (FB)
	PSF-based	FD and contrast (64B)	AUC	Variance, variance (FB), sum variance (FB), and autocorrelation (FB)	Skewness, cluster shade (FB), and cluster tendency (FB)	Skewness and cluster prominence (FB)	Cluster shade (64B and FB) and correlation (64B and FB)
CT-based	EANM-compliant	SRE and compactness A	AUC	Skewness, cluster shade (64B), autocorrelation (FB), cluster tendency (FB), contrast (FB), and sum variance (FB)	Cluster shade (FB) and cluster prominence (FB)	–	Skewness, cluster prominence (FB), and cluster shade (64B and FB)
	PSF-based	–	–	Skewness, autocorrelation (FB), contrast (FB), and sum variance (FB)	Cluster shade (FB) and cluster prominence (FB)	Cluster prominence (FB)	Skewness and cluster shade (FB)

Two types of SUV discretization were used, 64 grey-level bins (64B) or a fixed bin size of 0.25 g/ml (FB)

**Fig. 3:**
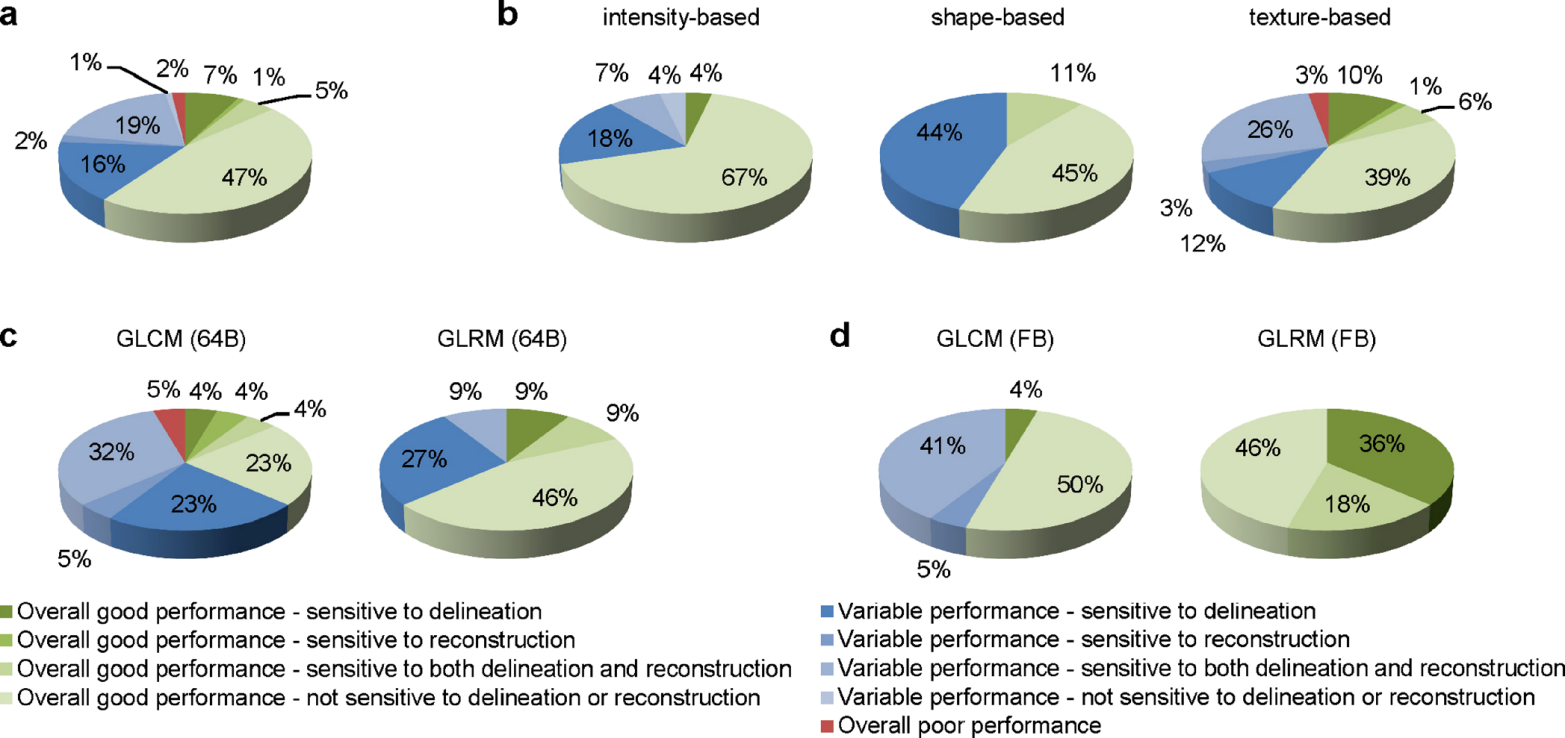
Performance of radiomic features extracted from EANM-compliant or PSF-based reconstructed PET images using PET-based or CT-based delineation. Performance is given for **a** all features; **b** intensity-based, shape-based, and texture-based features; **c** GLCM-based and GLRM-based features using 64 grey-level bins; and **d** GLCM-based and GLRM-based features using fixed bins.

**Fig. 4:**
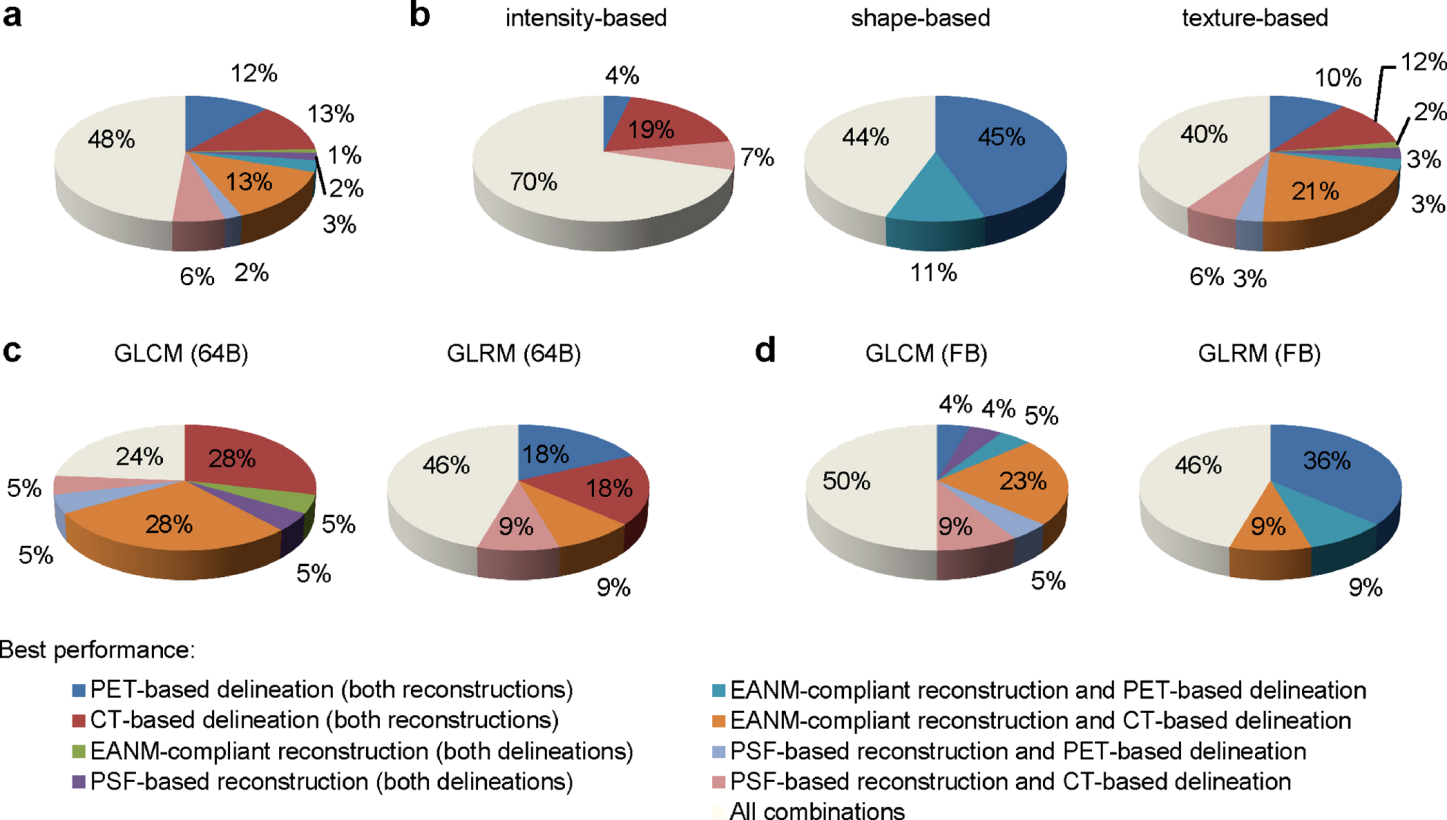
Combinations of delineation and reconstruction showing the best performance, given for **a** all features; **b** intensity-based, shape-based, and texture-based features; **c** GLCM-based and GLRM-based features using 64 grey-level bins; and **d** GLCM-based and GLRM-based features using fixed bins. Features that showed a poor performance were not included.

Sixty three out of 105 radiomic features showed a good performance (i.e., ICC ≥ 0.9) independent of the applied delineation or selected reconstruction algorithm, while 40 features only showed a good performance for certain combinations of reconstruction algorithm and/or delineation (Fig. 3a). More features were sensitive to a change in delineation than to a change in reconstruction (25 and 3 features, respectively), and 25 features were sensitive to a change in both reconstruction and delineation. Only fractal dimension and homogeneity 2 (obtained using 64 grey-level bins) showed an overall poor performance. After excluding these two features, the majority of the features showed less than 0.03 difference in ICC for either applied delineation and/or reconstruction (49 %; Fig. 4a). The best performance was seen using CT-based delineation (32 %), followed by EANM-compliant reconstruction or PET-based delineation (both 17 %), and PSF-based reconstruction (10 %).

More than two thirds of the intensity-based features (70 %) and one third of shape-based and texture-based features show an overall good performance (56 and 57 %, respectively; Fig. 3b). After excluding the features with an overall poor performance, most intensity-based features had a less than 0.03 difference in ICC for either applied delineation and/or reconstruction (70 %). Most shape-based features showed the best performance using PET-based delineation (56 %), while most texture-based features showed the best performance using CT-based delineation (39 %; Fig. 4b).

The percentages of both GLCM-based and GLRM-based features showing an overall good performance increased when fixed bins were applied compared to 64 grey-level bins (55 and 100 % vs 36 and 63 %, respectively; Fig. 3c, d). After excluding those features showing an overall poor performance, most features showed less than 0.03 difference in ICC for either applied delineation and/or reconstruction, except for GLCM-based features when 64 grey-level bins were applied, showing the best performance using CT-based delineation (62 %; Fig. 4c).

## Discussion

The present study shows that the majority of radiomic features show a high level of repeatability that is similar or better compared to simple SUV measures such as SUV_mean_ in terms of ICC, TRT_*r*_, and TRT_*a*_ [12, 32]. These results are in line with three previous studies by Leijenaar et al. [11], Tixier et al. [14], and Van Velden et al. [13], investigating the repeatability of various radiomic features in NSCLC patients, esophageal cancer patients, and patients with colorectal liver metastases, respectively. Data presented in these studies and the present study enable a preselection of well-performing features per category in order to further assess them for their clinical applicability.

To the best of our knowledge, this is the first study that investigates the impact of various reconstructions and delineations on the repeatability of several radiomic features, including intensity-, shape-, and texture-based features. However, this is not the first study that investigates the impact of reconstruction and delineation on radiomic features. A previous study by Hatt et al. [26] investigated the impact of reconstruction-based partial volume correction and various PET-based delineation on radiomic features in terms of therapy response prediction for esophageal cancer patients, showing that the performance of radiomic features were more dependent on delineation than on partial volume correction (i.e., reconstruction settings). Two studies [25, 27] investigated the effects of different reconstruction settings on the values obtained from various texture-based features. Galavis et al. [25] found that most features (80 %) showed a large variation between values (>30 %) when reconstruction settings were varied. Yan et al. [27] showed that 5 to 56 % of the features showed a large variation between values (>20 %) when reconstruction settings were varied and that zone percentage, cluster shade, and skewness should be used with caution. The level of features sensitive to the reconstruction settings is expected to be different in the present study, as the present study does not investigate differences between values obtained from features extracted from PSF-based and EANM-compliant reconstructed images but investigates whether or not they show repeatable results. Note that thresholds used in this study are arbitrary and only intended to illustrate which features are sensitive to delineation and/or reconstruction. Nonetheless, our study confirmed that many texture-based features (36 %) were sensitive to the selected reconstruction algorithm by showing a change in repeatability (i.e., showing a more than 3 % difference in ICC). In addition, we observed a large variation in repeatability for skewness and cluster shade when reconstruction settings were varied.

Recently, Leijenaar et al. [21] investigated the effects of SUV discretizations on radiomic features and concluded that the manner of SUV discretization (i.e., fixed bin size in units of SUV or a fixed number of bins) had a crucial impact on the values of various texture-based radiomic features and the interpretation thereof. They suggest that using a fixed bin size in units of SUV is more appropriate in a clinical response monitoring setting as it can incorporate changes in SUV due to a course of treatment. Our present study shows that using a fixed bin size in units of SUV results in texture-based features that show a better repeatability and a lower sensitivity to a change in delineation and/or reconstruction compared to using a fixed number of bins. A previous study [14] showed that 64 grey-level bins are best suited for extraction of radiomic features when a fixed number of bins is applied. This would, on average, translate to 0.25 g/ml for the lesions in the present study. However, a fixed bin size of 0.5 g/ml has been applied in a previous publication [11], but no further motivation is provided. A clinical study that includes outcome measures is required to validate which fixed bin size is optimal in a clinical setting. Nevertheless, this study confirms that, if a fixed bin size is best suited for clinical response monitoring, a standardized methodology in texture analysis is needed to compare results in a multicenter setting, i.e., by standardization of reconstruction settings, delineations, and SUV discretization [18, 21, 33].

A limitation of this study is that the CT-based delineation is obtained manually. Therefore, these results may to a small extent be affected by inter-observer variability [11, 34]. Ideally, the effects of inter-observer variability on our results should be assessed by manual CT delineation by three observers. In this study, we aimed to minimize the impact of inter-observer variability by achieving consensus by means of discussion between three experienced observers.

## Conclusion

In this paper, we report on the repeatability of radiomic features for NSCLC [^18^F]FDG-PET/CT studies, showing that many features have similar TRT and ICC performance as more commonly used PET parameters, such as SUV_max_, SUV_mean_, and SUV_peak_. Furthermore, PSF-based reconstructions do not necessarily result in improved repeatability of radiomic features when compared to EANM-compliant reconstructions. Performance of radiomic features depended more on delineation method than on the applied reconstruction algorithm. CT-based delineation showed favorable repeatabilities and ICCs for most radiomic features, except for shape-based features for which PET-based delineation resulted in better performance in terms of TRT and ICC.

## Electronic Supplementary Material

Below is the link to the electronic supplementary material.

ESM 1 (PDF 255 kb)
