# Supplementary material for: Repeatability of Radiomic Features in Non-Small-Cell Lung Cancer [18F]FDG-PET/CT Studies: Impact of Reconstruction and Delineation
Source: Mol Imaging Biol. 2016 Feb 26;18(5):788–95. doi: 10.1007/s11307-016-0940-2 (PMC5010602; doi:10.1007/s11307-016-0940-2)
Supplement: Supplementary file 1 — (PDF 255 kb) [file 11307_2016_940_MOESM1_ESM.pdf]

Supplemental data

Repeatability of radiomics features in non-small cell lung cancer FDG-PET/CT studies: impact of reconstruction and delineation

Journal: Molecular Imaging and Biology

Floris HP van Velden[1,2,*], Gerbrand M Kramer[1], Virginie Frings[1], Ida A Nissen[1], Emma R Mulder[1], Adrianus J de Langen[3], Otto S Hoekstra[1], Egbert F Smit[3,4], Ronald Boellaard[1*]

[1]*Department of Radiology & Nuclear Medicine, VU University Medical Center, Amsterdam, The Netherlands*
[2]*Division of Nuclear Medicine, Department of Radiology, Leiden University Medical Center, Leiden, The Netherlands*
[3]*Department of Pulmonary Diseases, VU University Medical Center, Amsterdam, The Netherlands*
[4]*Department of Thoracic Oncology, Netherlands Cancer Institute, Amsterdam, The Netherlands*
**presently affiliated with the Department of Nuclear Medicine and Molecular Imaging, University Medical Centre Groningen, Groningen, The Netherlands.*

[*]Corresponding author: Floris H.P. van Velden, PhD
VU University Medical Center
Department of Radiology & Nuclear Medicine
PO Box 7057
1007MB Amsterdam
The Netherlands
E-mail: f.h.p.van_velden@lumc.nl
Tel: +31(0)715265726 / Fax: +31(0)715264649

Supplemental Table 1. Intraclass correlation coefficients (ICC) and 95% confidence intervals (CI) of various intensity-based features.

	PET-based delineation						CT-based delineation					
	EANM-compliant reconstruction			PSF-based reconstruction			EANM-compliant reconstruction			PSF-based reconstruction		
		CI			CI			CI			CI	
Radiomics features*	ICC	Lower	Upper	ICC	Lower	Upper	ICC	Lower	Upper	ICC	Lower	Upper
TLG	0.997	0.993	0.999	0.992	0.981	0.997	0.999	0.999	1.000	0.999	0.999	1.000
Total energy	0.998	0.994	0.999	0.993	0.982	0.997	0.998	0.996	0.999	0.998	0.996	0.999
SUV_{min}	0.990	0.974	0.996	0.989	0.973	0.996	0.901	0.766	0.960	0.888	0.739	0.955
SUV_{median}	0.989	0.973	0.996	0.987	0.967	0.995	0.972	0.929	0.989	0.965	0.914	0.986
SUV_{peak}	0.989	0.971	0.996	0.986	0.965	0.995	0.989	0.971	0.996	0.986	0.965	0.995
SUV_{mean}	0.989	0.971	0.996	0.987	0.967	0.995	0.979	0.947	0.992	0.976	0.940	0.991
RMS	0.988	0.970	0.995	0.987	0.966	0.995	0.981	0.953	0.993	0.979	0.947	0.992
Variance	0.985	0.961	0.994	0.986	0.964	0.994	0.979	0.946	0.992	0.978	0.945	0.991
SUV_{max}	0.983	0.957	0.993	0.979	0.948	0.992	0.983	0.957	0.993	0.979	0.948	0.992
SUV_{range}	0.972	0.930	0.989	0.965	0.913	0.986	0.981	0.952	0.993	0.977	0.943	0.991
SD	0.973	0.932	0.989	0.970	0.926	0.988	0.978	0.945	0.991	0.976	0.940	0.991
Median absolute deviation	0.972	0.929	0.989	0.968	0.921	0.988	0.976	0.939	0.990	0.975	0.938	0.990
Mean absolute deviation	0.972	0.929	0.989	0.969	0.922	0.988	0.975	0.938	0.990	0.974	0.936	0.990
Laplacian mean	0.975	0.937	0.990	0.970	0.925	0.988	0.952	0.882	0.981	0.950	0.877	0.980
SUV_{star}	0.971	0.927	0.988	0.974	0.934	0.990	0.971	0.927	0.989	0.974	0.934	0.990
Skewness	0.908	0.782	0.963	0.929	0.829	0.972	0.963	0.909	0.986	0.969	0.923	0.988
Entropy (FB)	0.965	0.913	0.986	0.965	0.913	0.986	0.969	0.922	0.988	0.962	0.906	0.985
Kurtosis	0.876	0.712	0.950	0.904	0.773	0.962	0.960	0.902	0.984	0.967	0.919	0.987
Uniformity (FB)	0.966	0.916	0.987	0.964	0.911	0.986	0.967	0.919	0.987	0.966	0.915	0.987
Local entropy (FB)	0.962	0.906	0.985	0.953	0.884	0.981	0.954	0.887	0.982	0.949	0.875	0.980
Geary's C	0.939	0.851	0.976	0.940	0.853	0.976	0.959	0.898	0.984	0.955	0.888	0.982
COV	0.845	0.648	0.937	0.893	0.749	0.957	0.938	0.850	0.976	0.934	0.840	0.974
Entropy (64B)	0.862	0.682	0.944	0.883	0.726	0.953	0.902	0.767	0.961	0.937	0.847	0.975
Local entropy (64B)	0.896	0.754	0.958	0.900	0.764	0.960	0.930	0.831	0.972	0.925	0.820	0.970
AUC	0.162	-0.294	0.563	0.534	0.128	0.789	0.757	0.479	0.898	0.913	0.794	0.965
Uniformity (64B)	0.862	0.682	0.944	0.880	0.721	0.952	0.902	0.768	0.961	0.903	0.770	0.961
Moran's I	0.902	0.769	0.961	0.887	0.735	0.955	0.887	0.735	0.954	0.890	0.743	0.956

*Two types of SUV discretization were applied: 64 grey level bins (64B) or a fixed bin size of 0.25 g/mL (FB). The features are sorted by the highest ICC obtained for any combination of reconstruction and delineation.

Supplemental Table 2. Intraclass correlation coefficients (ICC) and 95% confidence intervals (CI) of various shape-based and texture-based (fractal) features.

	PET-based delineation						CT-based delineation					
	EANM-compliant reconstruction			PSF-based reconstruction			EANM-compliant reconstruction			PSF-based reconstruction		
		CI			CI			CI			CI	
Radiomics features*	ICC	Lower	Upper	ICC	Lower	Upper	ICC	Lower	Upper	ICC	Lower	Upper
Compactness A	0.983	0.957	0.993	0.969	0.923	0.988	0.999	0.997	1.000	0.999	0.997	1.000
Radius of an equivolumetric sphere	0.996	0.990	0.998	0.993	0.982	0.997	0.999	0.997	0.999	0.999	0.997	0.999
Surface area	0.998	0.994	0.999	0.999	0.996	0.999	0.996	0.989	0.998	0.996	0.989	0.998
MATV/AV	0.991	0.978	0.997	0.983	0.957	0.993	0.997	0.993	0.999	0.997	0.993	0.999
Abundance	0.985	0.962	0.994	0.988	0.971	0.995	0.994	0.985	0.998	0.994	0.985	0.998
S2V	0.982	0.954	0.993	0.938	0.849	0.975	0.980	0.950	0.992	0.980	0.950	0.992
Compactness B	0.964	0.911	0.986	0.980	0.950	0.992	0.849	0.656	0.939	0.849	0.656	0.939
Sphericity	0.962	0.907	0.985	0.978	0.945	0.991	0.870	0.699	0.947	0.870	0.699	0.947
$S2V_{eq}$	0.946	0.869	0.979	0.965	0.913	0.986	0.886	0.733	0.954	0.886	0.733	0.954
Disproportion	0.944	0.863	0.978	0.964	0.910	0.986	0.887	0.736	0.955	0.887	0.736	0.955
Lacunarity	0.911	0.787	0.964	0.816	0.591	0.924	0.916	0.800	0.967	0.916	0.800	0.967
FD	0.740	0.448	0.890	0.686	0.358	0.865	0.877	0.714	0.950	0.877	0.714	0.950

*The features are sorted by the highest ICC obtained for any combination of reconstruction and delineation.

Supplemental Table 3. Intraclass correlation coefficients (ICC) and 95% confidence intervals (CI) of various texture-based features (based on grey-level co-occurrence matrices).

	PET-based delineation						CT-based delineation					
	EANM-compliant reconstruction			PSF-based reconstruction			EANM-compliant reconstruction			PSF-based reconstruction		
		CI			CI			CI			CI	
**Radiomics features***	**ICC**	**Lower**	**Upper**	**ICC**	**Lower**	**Upper**	**ICC**	**Lower**	**Upper**	**ICC**	**Lower**	**Upper**
Cluster prominence (FB)	0.995	0.988	0.998	0.994	0.985	0.998	0.992	0.979	0.997	0.992	0.979	0.997
Cluster shade (FB)	0.948	0.873	0.979	0.860	0.679	0.943	0.994	0.984	0.998	0.993	0.982	0.997
Inverse variance (64B)	0.991	0.978	0.997	0.989	0.973	0.996	0.983	0.957	0.993	0.982	0.954	0.993
IMC1 (64B)	0.982	0.954	0.993	0.986	0.965	0.995	0.979	0.947	0.992	0.991	0.977	0.996
Autocorrelation (FB)	0.988	0.970	0.995	0.984	0.961	0.994	0.990	0.974	0.996	0.989	0.972	0.996
Sum variance (FB)	0.989	0.972	0.996	0.987	0.967	0.995	0.989	0.973	0.996	0.989	0.971	0.996
Variance (FB)	0.989	0.972	0.996	0.988	0.969	0.995	0.989	0.971	0.996	0.988	0.969	0.995
Contrast (FB)	0.987	0.968	0.995	0.987	0.967	0.995	0.974	0.935	0.990	0.977	0.941	0.991
IMC2 (64B)	0.965	0.912	0.986	0.975	0.937	0.990	0.954	0.886	0.982	0.984	0.960	0.994
Sum average (FB)	0.983	0.957	0.993	0.981	0.951	0.992	0.983	0.958	0.994	0.982	0.955	0.993
Entropy (64B)	0.954	0.887	0.982	0.945	0.865	0.978	0.983	0.956	0.993	0.983	0.956	0.993
Cluster tendency (FB)	0.981	0.953	0.993	0.980	0.949	0.992	0.972	0.929	0.989	0.969	0.923	0.988
Entropy (FB)	0.955	0.890	0.982	0.960	0.902	0.984	0.976	0.941	0.991	0.981	0.952	0.993
Dissimilarity (FB)	0.971	0.928	0.989	0.979	0.947	0.992	0.935	0.843	0.974	0.941	0.856	0.977
Dissimilarity (64B)	0.944	0.864	0.978	0.858	0.674	0.942	0.973	0.933	0.990	0.972	0.929	0.989
IDN (64B)	0.957	0.893	0.983	0.881	0.723	0.952	0.973	0.932	0.989	0.971	0.928	0.989
IDMN (64B)	0.921	0.811	0.969	0.817	0.591	0.925	0.971	0.929	0.989	0.967	0.919	0.987
IDMN (FB)	0.914	0.796	0.966	0.820	0.597	0.926	0.969	0.924	0.988	0.967	0.918	0.987
IDN (FB)	0.945	0.866	0.978	0.862	0.683	0.944	0.968	0.920	0.987	0.969	0.923	0.988
Correlation (FB)	0.819	0.596	0.926	0.915	0.797	0.966	0.968	0.920	0.987	0.967	0.918	0.987
Contrast (64B)	0.882	0.724	0.952	0.772	0.507	0.905	0.967	0.918	0.987	0.958	0.896	0.983
Correlation (64B)	0.800	0.559	0.917	0.915	0.797	0.966	0.967	0.918	0.987	0.966	0.915	0.987
Sum entropy (FB)	0.947	0.869	0.979	0.959	0.898	0.984	0.963	0.909	0.986	0.962	0.907	0.985
IMC1 (FB)	0.960	0.902	0.984	0.954	0.888	0.982	0.962	0.906	0.985	0.943	0.860	0.977
Difference entropy (64B)	0.891	0.743	0.956	0.908	0.780	0.963	0.960	0.902	0.984	0.959	0.899	0.984
Sum average (64B)	0.937	0.847	0.975	0.924	0.816	0.970	0.956	0.891	0.983	0.944	0.863	0.978
Sum entropy (64B)	0.874	0.708	0.949	0.918	0.803	0.967	0.954	0.887	0.982	0.945	0.866	0.978
Difference entropy (FB)	0.935	0.842	0.974	0.953	0.885	0.981	0.934	0.840	0.974	0.941	0.856	0.977
Inverse variance (FB)	0.950	0.877	0.980	0.897	0.758	0.959	0.936	0.846	0.975	0.943	0.860	0.977
Autocorrelation (64B)	0.937	0.846	0.975	0.907	0.779	0.963	0.949	0.875	0.980	0.924	0.817	0.970
Maximum probability (FB)	0.896	0.755	0.958	0.934	0.840	0.974	0.949	0.874	0.980	0.947	0.871	0.979
Maximum probability (64B)	0.897	0.757	0.959	0.930	0.830	0.972	0.946	0.869	0.979	0.946	0.868	0.979
IMC2 (FB)	0.898	0.760	0.959	0.900	0.764	0.960	0.946	0.867	0.979	0.915	0.797	0.966
Cluster shade (64B)	0.913	0.793	0.965	0.945	0.866	0.978	0.933	0.839	0.974	0.914	0.794	0.966
Sum variance (64B)	0.908	0.782	0.963	0.882	0.725	0.952	0.945	0.865	0.978	0.918	0.803	0.967
Variance (64B)	0.880	0.721	0.952	0.865	0.688	0.945	0.943	0.862	0.978	0.919	0.806	0.968
Cluster tendency (64B)	0.920	0.807	0.968	0.932	0.835	0.973	0.942	0.859	0.977	0.938	0.850	0.975
Homogeneity 1 (FB)	0.901	0.766	0.960	0.935	0.843	0.974	0.892	0.747	0.957	0.905	0.774	0.962
Energy (64B)	0.856	0.669	0.941	0.916	0.800	0.967	0.933	0.839	0.974	0.934	0.840	0.974
Energy (FB)	0.887	0.735	0.954	0.927	0.824	0.971	0.924	0.817	0.970	0.929	0.830	0.972
Homogeneity 2 (FB)	0.896	0.755	0.958	0.927	0.824	0.971	0.897	0.756	0.959	0.907	0.780	0.963
Cluster prominence (64B)	0.902	0.768	0.961	0.895	0.753	0.958	0.910	0.786	0.964	0.878	0.715	0.951
Homogeneity 1 (64B)	0.767	0.497	0.903	0.854	0.665	0.941	0.903	0.770	0.961	0.897	0.757	0.959
Homogeneity 2 (64B)	0.779	0.520	0.908	0.867	0.694	0.946	0.881	0.723	0.952	0.876	0.712	0.950

*Two types of SUV discretization were applied: 64 grey level bins (64B) or a fixed bin size of 0.25 g/mL (FB). The features are sorted by the highest ICC obtained for any combination of reconstruction and delineation.

Supplemental Table 4. Intraclass correlation coefficients (ICC) and 95% confidence intervals (CI) of various texture-based features (based on grey-level run-length matrices).

	PET-based delineation						CT-based delineation					
	EANM-compliant reconstruction			PSF-based reconstruction			EANM-compliant reconstruction			PSF-based reconstruction		
		CI			CI			CI			CI	
**Radiomics features***	**ICC**	**Lower**	**Upper**	**ICC**	**Lower**	**Upper**	**ICC**	**Lower**	**Upper**	**ICC**	**Lower**	**Upper**
RLN (FB)	0.992	0.980	0.997	0.987	0.968	0.995	0.999	0.997	0.999	0.999	0.998	1.000
RLN (64B)	0.994	0.985	0.998	0.991	0.977	0.996	0.987	0.967	0.995	0.996	0.990	0.998
RP (64B)	0.992	0.979	0.997	0.988	0.969	0.995	0.989	0.972	0.996	0.986	0.965	0.995
GLN (FB)	0.992	0.979	0.997	0.991	0.976	0.996	0.953	0.885	0.982	0.951	0.880	0.981
LRHGLE (FB)	0.991	0.976	0.996	0.988	0.971	0.995	0.991	0.978	0.997	0.990	0.976	0.996
HGLRE (FB)	0.988	0.971	0.995	0.987	0.967	0.995	0.990	0.974	0.996	0.990	0.974	0.996
SRHGLE (FB)	0.988	0.969	0.995	0.987	0.967	0.995	0.989	0.973	0.996	0.990	0.974	0.996
GLN (64B)	0.989	0.972	0.996	0.986	0.966	0.995	0.920	0.808	0.968	0.929	0.828	0.972
SRLGLE (64B)	0.983	0.956	0.993	0.958	0.896	0.983	0.981	0.952	0.993	0.981	0.953	0.993
SRLGLE (FB)	0.981	0.953	0.993	0.960	0.900	0.984	0.981	0.952	0.993	0.981	0.952	0.993
RP (FB)	0.978	0.946	0.992	0.979	0.948	0.992	0.934	0.840	0.974	0.951	0.880	0.981
LRE (FB)	0.969	0.924	0.988	0.938	0.850	0.976	0.918	0.804	0.967	0.926	0.822	0.971
SRE (FB)	0.968	0.919	0.987	0.950	0.878	0.980	0.934	0.841	0.974	0.923	0.816	0.969
LGLRE (FB)	0.963	0.908	0.985	0.916	0.798	0.966	0.965	0.912	0.986	0.964	0.912	0.986
LRLGLE (FB)	0.963	0.909	0.986	0.934	0.841	0.974	0.910	0.786	0.964	0.920	0.808	0.968
LRE (64B)	0.959	0.900	0.984	0.944	0.864	0.978	0.938	0.850	0.976	0.940	0.854	0.976
LGLRE (64B)	0.949	0.876	0.980	0.911	0.788	0.964	0.954	0.887	0.982	0.959	0.898	0.984
LRLGLE (64B)	0.958	0.897	0.984	0.943	0.861	0.978	0.934	0.840	0.974	0.936	0.844	0.975
LRHGLE (64B)	0.905	0.775	0.962	0.842	0.642	0.936	0.951	0.879	0.981	0.922	0.813	0.969
SRE (64B)	0.927	0.825	0.971	0.907	0.779	0.963	0.847	0.651	0.938	0.858	0.674	0.942
HGLRE (64B)	0.872	0.703	0.948	0.814	0.586	0.923	0.925	0.819	0.970	0.895	0.753	0.958
SRHGLE (64B)	0.867	0.694	0.946	0.809	0.576	0.921	0.918	0.803	0.967	0.889	0.740	0.956

*Two types of SUV discretization were applied: 64 grey level bins (64B) or a fixed bin size of 0.25 g/mL (FB). The features are sorted by the highest ICC obtained for any combination of reconstruction and delineation.

Supplemental Table 5. Mean relative test-retest variability (TRT_r, %) and 95% confidence intervals (CI) of various intensity-based features.

	PET-based delineation						CT-based delineation					
	EANM-compliant reconstruction			PSF-based reconstruction			EANM-compliant reconstruction			PSF-based reconstruction		
		CI			CI			CI			CI	
**Radiomics features***	**TRT_r**	**Lower**	**Upper**	**TRT_r**	**Lower**	**Upper**	**TRT_r**	**Lower**	**Upper**	**TRT_r**	**Lower**	**Upper**
Local entropy (64B)	-0.3	-2.8	2.2	0.4	-2.0	2.9	-0.1	-2.2	2.1	0.1	-2.2	2.4
Entropy (64B)	0.6	-3.0	4.1	0.5	-4.0	4.9	-0.4	-3.8	2.9	-0.2	-3.3	3.0
Local entropy (FB)	0.3	-4.8	5.4	1.1	-2.4	4.7	1.0	-3.4	5.3	0.8	-2.8	4.4
Entropy (FB)	2.5	-7.3	12.4	2.7	-5.4	10.9	2.3	-5.3	9.9	2.0	-5.6	9.6
Geary's C	0.0	-13.8	13.8	1.2	-9.2	11.5	4.8	-23.3	32.9	4.9	-22.2	31.9
COV	2.5	-12.4	17.4	2.7	-11.9	17.4	-0.2	-20.0	19.6	0.0	-19.5	19.5
Kurtosis	0.8	-21.2	22.9	0.8	-23.9	25.6	1.9	-13.2	17.0	2.8	-12.1	17.6
Uniformity (64B)	-2.1	-17.3	13.2	-0.9	-19.2	17.3	1.8	-15.0	18.5	0.2	-19.5	19.8
Moran's I	1.3	-23.7	26.3	-2.3	-29.3	24.7	-5.5	-22.2	11.2	-6.1	-26.3	14.1
SUV_{median}	4.4	-13.8	22.7	5.2	-16.8	27.2	5.8	-18.8	30.4	6.4	-20.1	32.9
AUC	0.9	-22.3	24.0	1.0	-17.2	19.3	2.1	-26.1	30.3	1.7	-19.6	23.0
SUV_{min}	4.1	-14.5	22.8	4.3	-18.9	27.5	-6.6	-53.2	40.1	-8.4	-62.2	45.5
SUV_{mean}	4.6	-14.7	24.0	5.1	-16.9	27.0	5.4	-17.0	27.8	5.5	-17.5	28.5
RMS	4.7	-14.8	24.3	5.2	-16.8	27.1	5.3	-16.1	26.6	5.4	-16.5	27.3
SUV_{peak}	4.9	-17.9	27.8	4.7	-20.8	30.2	4.9	-17.9	27.8	4.6	-20.8	30.0
Laplacian mean	6.2	-17.3	29.8	5.8	-23.9	35.5	6.8	-23.6	37.3	7.1	-24.0	38.2
SUV_{max}	4.7	-19.4	28.8	5.5	-20.7	31.8	4.7	-19.5	28.9	5.5	-20.7	31.7
TLG	7.5	-19.8	34.9	4.9	-22.1	31.8	5.3	-19.0	29.6	5.4	-19.0	29.8
Median absolute deviation	6.7	-22.5	35.9	7.1	-20.5	34.7	5.1	-19.2	29.4	5.2	-19.8	30.3
Mean absolute deviation	6.7	-22.5	35.8	7.3	-20.4	34.9	4.9	-19.4	29.3	5.0	-19.9	29.9
SD	7.1	-21.8	36.0	7.8	-19.2	34.8	5.2	-19.3	29.7	5.5	-19.4	30.4
Uniformity (FB)	-6.3	-33.0	20.4	-7.5	-33.0	18.1	-6.8	-34.0	20.4	-6.5	-39.4	26.3
SUV_{range}	5.2	-26.5	37.0	6.5	-24.8	37.8	7.3	-21.7	36.4	7.3	-22.5	37.1
SUV_{star}	3.6	-32.7	39.9	3.9	-26.7	34.5	3.1	-32.8	39.0	3.7	-26.9	34.2
Total energy	12.2	-31.1	55.4	10.0	-33.2	53.2	10.3	-32.9	53.6	10.5	-33.5	54.5
Variance	13.9	-42.8	70.6	15.2	-37.3	67.8	10.2	-38.1	58.6	10.8	-38.4	60.0
Skewness	0.4	-57.1	58.0	23.1	-149	195	-13.1	-212	186	-14.9	-161	131

*Two types of SUV discretization were applied: 64 grey level bins (64B) or a fixed bin size of 0.25 g/mL (FB). The features are sorted by the lowest absolute difference between upper and lower CI obtained for any combination of reconstruction and delineation.

Supplemental Table 6. Mean relative test-retest variability (TRT_r, %) and 95% confidence intervals (CI) of various shape-based and texture-based (fractal) features.

	**PET-based delineation**						**CT-based delineation**					
	**EANM-compliant reconstruction**			**PSF-based reconstruction**			**EANM-compliant reconstruction**			**PSF-based reconstruction**		
		**CI**			**CI**			**CI**			**CI**	
**Radiomics features***	**TRT_r**	**Lower**	**Upper**	**TRT_r**	**Lower**	**Upper**	**TRT_r**	**Lower**	**Upper**	**TRT_r**	**Lower**	**Upper**
Abundance	1.3	-4.4	7.0	0.3	-5.0	5.6	0.3	-3.4	3.9	0.3	-3.4	3.9
Radius of an equivolumetric sphere	1.0	-5.5	7.4	-0.1	-7.8	7.7	0.0	-4.5	4.5	0.0	-4.5	4.5
FD	2.1	-14.2	18.3	2.3	-14.0	18.5	0.7	-8.3	9.7	0.7	-8.3	9.7
Compactness A	1.3	-11.7	14.3	0.7	-15.3	16.7	-0.3	-10.0	9.4	-0.3	-10.0	9.4
$S2V_{eq}$	0.5	-9.8	10.7	-1.2	-12.2	9.9	0.4	-11.1	11.9	0.4	-11.1	11.9
Sphericity	-0.5	-10.7	9.8	1.1	-9.9	12.2	-0.4	-11.8	11.1	-0.4	-11.8	11.1
Disproportion	0.5	-9.8	10.7	-1.1	-12.2	9.9	0.4	-11.1	11.8	0.4	-11.1	11.8
S2V	-0.5	-12.8	11.8	-1.1	-15.7	13.6	0.4	-11.2	12.0	0.4	-11.2	12.0
MATV/AV	2.9	-16.5	22.3	-0.2	-23.3	22.9	-0.1	-13.6	13.4	-0.1	-13.6	13.4
Lacunarity	3.2	-15.6	22.0	3.8	-18.8	26.5	1.6	-12.6	15.9	1.6	-12.6	15.9
Surface area	2.4	-13.8	18.5	-1.3	-18.4	15.8	0.3	-15.5	16.1	0.3	-15.5	16.1
Compactness B	-1.4	-31.9	29.1	3.4	-29.6	36.4	-1.1	-35.4	33.2	-1.1	-35.4	33.2

* The features are sorted by the lowest absolute difference between upper and lower CI obtained for any combination of reconstruction and delineation.

Supplemental Table 7. Mean relative test-retest variability (TRT_r, %) and 95% confidence intervals (CI) of various texture-based features (based on grey-level co-occurrence matrices).

	**PET-based delineation**						**CT-based delineation**					
	**EANM-compliant reconstruction**			**PSF-based reconstruction**			**EANM-compliant reconstruction**			**PSF-based reconstruction**		
		**CI**			**CI**			**CI**			**CI**	
**Radiomics features***	**TRT_r**	**Lower**	**Upper**	**TRT_r**	**Lower**	**Upper**	**TRT_r**	**Lower**	**Upper**	**TRT_r**	**Lower**	**Upper**
IDMN (FB)	0.0	-1.7	1.6	0.1	-2.0	2.1	-0.1	-1.5	1.2	0.0	-1.2	1.2
IDMN (64B)	0.0	-1.9	1.9	0.1	-1.9	2.1	-0.1	-1.5	1.3	0.0	-1.2	1.2
IDN (64B)	-0.1	-2.0	1.8	0.0	-2.2	2.3	-0.3	-2.1	1.4	-0.2	-1.9	1.4
IDN (FB)	-0.1	-1.9	1.7	0.0	-2.4	2.3	-0.4	-2.1	1.4	-0.3	-1.9	1.4
IMC2 (64B)	0.4	-4.3	5.1	0.9	-4.2	6.0	0.7	-2.3	3.6	0.4	-1.6	2.5
Sum entropy (64B)	0.9	-4.0	5.9	0.6	-4.7	5.8	0.7	-1.9	3.4	0.7	-2.1	3.5
Entropy (64B)	0.9	-5.0	6.9	0.7	-6.4	7.7	0.8	-2.3	3.9	0.8	-2.3	3.9
Difference entropy (64B)	1.1	-3.0	5.1	0.7	-3.9	5.3	0.6	-2.8	4.1	0.5	-2.9	3.9
IMC1 (64B)	0.5	-11.6	12.5	1.9	-10.6	14.3	1.6	-7.5	10.7	0.8	-5.6	7.3
Entropy (FB)	1.7	-9.8	13.2	2.0	-6.6	10.6	2.6	-5.6	10.7	2.1	-4.5	8.6
Sum entropy (FB)	1.6	-9.5	12.8	2.0	-5.6	9.6	2.7	-5.1	10.6	2.3	-4.6	9.2
Difference entropy (FB)	2.4	-9.8	14.6	2.4	-6.5	11.3	3.3	-6.0	12.6	2.6	-5.4	10.7
IMC2 (FB)	0.9	-11.5	13.3	2.3	-8.5	13.1	2.2	-6.4	10.9	2.3	-5.8	10.4
Dissimilarity (64B)	0.4	-9.3	10.2	-0.6	-12.3	11.1	1.5	-10.8	13.9	1.1	-11.5	13.6
Sum average (64B)	0.1	-10.3	10.5	0.1	-11.6	11.8	1.9	-9.8	13.6	1.2	-11.6	14.1
Homogeneity 1 (64B)	-1.1	-22.4	20.1	-1.0	-22.2	20.2	-4.9	-15.4	5.6	-4.6	-15.5	6.4
Homogeneity 2 (64B)	-1.2	-29.8	27.5	-1.2	-29.5	27.0	-7.1	-21.7	7.5	-6.7	-22.5	9.1
Inverse variance (64B)	-1.2	-17.6	15.1	-0.3	-16.4	15.8	-3.2	-25.9	19.4	-4.3	-29.3	20.8
Cluster tendency (64B)	1.5	-19.4	22.4	-0.5	-17.0	15.9	-0.2	-19.7	19.4	-1.2	-21.8	19.4
IMC1 (FB)	0.5	-17.6	18.6	3.7	-18.0	25.4	3.8	-12.8	20.4	5.4	-13.6	24.4
Variance (64B)	0.1	-17.2	17.4	-0.2	-19.0	18.7	2.4	-17.7	22.5	1.1	-20.5	22.7
Homogeneity 1 (FB)	-2.1	-20.1	15.9	-3.3	-20.7	14.1	-6.9	-24.4	10.5	-7.1	-24.7	10.6
Sum variance (64B)	0.1	-18.2	18.4	0.0	-20.0	20.0	2.6	-18.8	24.0	1.2	-21.9	24.2
Contrast (64B)	0.2	-18.3	18.7	-1.5	-22.0	19.0	1.2	-23.4	25.8	0.2	-25.3	25.7
Autocorrelation (64B)	0.2	-18.3	18.7	0.4	-20.3	21.1	2.8	-18.0	23.6	1.5	-20.9	23.8
Maximum probability (FB)	2.2	-39.0	43.4	-1.5	-40.8	37.8	-10.2	-32.7	12.3	-10.5	-31.4	10.3
Maximum probability (64B)	0.7	-44.3	45.7	-0.3	-42.8	42.1	-10.7	-31.7	10.3	-10.3	-31.5	10.9
Homogeneity 2 (FB)	-2.5	-26.3	21.4	-3.7	-27.2	19.7	-9.2	-31.9	13.4	-9.4	-32.0	13.2
Sum average (FB)	4.3	-20.4	29.0	6.2	-17.1	29.4	8.7	-15.6	33.0	7.9	-16.0	31.7
Dissimilarity (FB)	5.0	-23.3	33.2	6.1	-21.5	33.7	9.4	-17.0	35.8	8.4	-17.7	34.5
Inverse variance (FB)	-4.5	-30.8	21.9	-5.1	-37.9	27.6	-9.6	-42.0	22.7	-10.4	-50.0	29.2
Correlation (FB)	4.6	-50.2	59.3	-8.4	-200	183	-2.2	-30.9	26.5	-1.9	-30.4	26.6
Correlation (64B)	6.9	-52.6	66.3	-6.7	-238	224	-2.0	-32.5	28.4	-2.0	-31.2	27.2
Energy (64B)	-1.6	-55.3	52.1	-1.8	-51.1	47.5	-9.1	-40.4	22.3	-9.1	-39.5	21.3
Cluster prominence (64B)	3.0	-34.5	40.5	0.1	-31.1	31.3	-1.1	-38.0	35.9	-2.7	-42.8	37.5
Energy (FB)	-2.4	-46.9	42.1	-2.6	-43.7	38.5	-10.4	-46.1	25.2	-10.0	-44.4	24.4
Autocorrelation (FB)	8.6	-40.8	58.1	12.2	-34.0	58.4	16.5	-31.3	64.4	14.9	-32.1	61.9
Variance (FB)	8.9	-41.2	58.9	12.2	-34.5	58.9	16.5	-30.7	63.7	14.8	-31.6	61.3
Contrast (FB)	9.3	-43.7	62.4	11.5	-40.8	63.7	16.8	-31.0	64.5	14.7	-33.3	62.8
Sum variance (FB)	9.6	-43.8	63.1	13.1	-36.6	62.8	17.7	-33.2	68.7	15.9	-33.8	65.6
Cluster tendency (FB)	10.3	-46.9	67.5	12.0	-39.9	63.9	15.3	-36.1	66.8	13.6	-37.6	64.9
Cluster shade (64B)	40.9	-420	502	-0.1	-158	158	22.0	-160	204	0.8	-58.5	60.1
Cluster prominence (FB)	19.7	-86.5	126	24.0	-73.2	121	27.9	-68.8	125	25.1	-73.0	123
Cluster shade (FB)	25.8	-158	210	-47.1	-651	556	31.3	-108	171	18.6	-79.2	117

*Two types of SUV discretization were applied: 64 grey level bins (64B) or a fixed bin size of 0.25 g/mL (FB). The features are sorted by the lowest absolute difference between upper and lower CI obtained for any combination of reconstruction and delineation.

Supplemental Table 8. Mean relative test-retest variability (TRT_r, %) and 95% confidence intervals (CI) of various texture-based features (based on grey-level run-length matrices).

	PET-based delineation						CT-based delineation					
	EANM-compliant reconstruction			PSF-based reconstruction			EANM-compliant reconstruction			PSF-based reconstruction		
		CI			CI			CI			CI	
Radiomics features*	TRT_r	Lower	Upper	TRT_r	Lower	Upper	TRT_r	Lower	Upper	TRT_r	Lower	Upper
SRE (64B)	-1.4	-10.0	7.2	1.5	-9.0	12.1	1.1	-8.4	10.6	0.8	-8.5	10.1
SRE (FB)	-1.6	-11.6	8.4	2.6	-9.0	14.2	1.3	-8.6	11.2	1.4	-8.5	11.3
LGLRE (FB)	2.8	-7.6	13.2	-1.0	-13.1	11.2	-2.4	-16.2	11.4	-2.5	-15.7	10.7
LGLRE (64B)	3.0	-8.0	14.0	-0.3	-12.8	12.1	-2.8	-16.3	10.8	-2.2	-14.5	10.1
SRLGLE (64B)	2.8	-12.9	18.6	4.7	-15.0	24.3	-3.9	-27.4	19.6	-3.6	-25.0	17.8
RLN (64B)	1.9	-17.4	21.2	0.9	-24.7	26.4	2.2	-16.1	20.5	1.0	-14.9	16.8
SRLGLE (FB)	2.6	-14.2	19.3	4.1	-15.3	23.4	-3.8	-25.1	17.6	-3.8	-25.2	17.7
RLN (FB)	2.2	-20.5	24.8	2.6	-25.3	30.4	1.7	-16.2	19.6	1.9	-14.9	18.8
SRHGLE (64B)	-2.0	-23.3	19.4	0.3	-24.8	25.5	2.6	-21.1	26.3	1.1	-23.8	25.9
RP (64B)	3.8	-18.6	26.1	0.9	-21.1	22.9	-0.6	-22.1	20.8	-1.2	-23.0	20.7
HGLRE (64B)	-2.1	-23.7	19.5	0.3	-24.8	25.5	2.4	-21.1	25.9	0.9	-23.6	25.5
LRHGLE (64B)	-2.4	-24.3	19.5	0.2	-24.5	24.9	1.5	-21.1	24.1	0.5	-22.6	23.5
GLN (FB)	9.5	-17.0	36.1	-3.2	-39.2	32.8	-5.3	-37.3	26.7	-5.2	-38.1	27.6
RP (FB)	-0.5	-35.0	34.1	-5.1	-40.0	29.8	-8.9	-39.2	21.4	-8.4	-41.2	24.3
GLN (64B)	10.2	-20.4	40.8	-2.7	-41.3	35.9	-5.3	-38.6	28.0	-5.0	-38.1	28.1
LRE (FB)	7.8	-30.4	45.9	-5.7	-49.8	38.3	-4.2	-47.6	39.1	-4.2	-47.3	38.9
LRHGLE (FB)	5.2	-33.3	43.7	9.7	-29.0	48.3	13.7	-26.7	54.1	12.6	-30.9	56.2
LRE (64B)	8.2	-31.7	48.1	-5.1	-49.7	39.6	-4.4	-46.7	37.9	-3.9	-46.4	38.5
LRLGLE (FB)	9.1	-34.9	53.1	-5.8	-54.2	42.5	-4.4	-51.3	42.5	-4.4	-50.9	42.1
LRLGLE (64B)	9.5	-35.4	54.4	-5.2	-53.8	43.4	-4.7	-50.4	40.9	-4.3	-50.0	41.5
HGLRE (FB)	6.4	-43.9	56.8	13.5	-31.7	58.6	16.8	-32.0	65.7	15.2	-33.3	63.7
SRHGLE (FB)	6.2	-48.6	61.1	14.2	-32.4	60.8	17.4	-32.9	67.7	15.9	-33.4	65.2

*Two types of SUV discretization were applied: 64 grey level bins (64B) or a fixed bin size of 0.25 g/mL (FB). The features are sorted by the lowest absolute difference between upper and lower CI obtained for any combination of reconstruction and delineation.

Supplemental Table 9. Mean absolute test-retest variability (TRT_a, %) and standard deviation (SD) of various intensity-based features.

	PET-based delineation				CT-based delineation			
	EANM-compliant reconstruction		PSF-based reconstruction		EANM-compliant reconstruction		PSF-based reconstruction	
Radiomics features*	Mean	SD	Mean	SD	Mean	SD	Mean	SD
Local entropy (64B)	1.0	0.8	1.0	0.8	0.8	0.7	0.8	0.8
Entropy (64B)	1.5	1.1	2.0	1.1	1.4	1.1	1.2	1.0
Local entropy (FB)	2.1	1.4	1.7	1.2	1.8	1.5	1.5	1.3
Entropy (FB)	4.1	3.8	4.1	2.7	3.6	2.6	3.3	2.7
Geary's C	5.3	4.5	4.4	2.9	9.7	11.4	9.7	10.8
AUC	7.8	8.7	5.8	7.3	10.7	9.5	8.1	7.2
COV	5.9	5.3	6.2	4.9	7.8	6.2	7.5	6.2
Uniformity (64B)	6.0	5.2	8.3	4.0	7.0	4.9	7.0	7.0
Kurtosis	8.1	7.6	10.4	6.8	6.9	3.5	6.7	4.2
SUV_{median}	6.8	7.7	8.6	8.7	8.9	10.5	9.3	11.6
SUV_{min}	7.0	7.6	9.0	8.5	18.7	15.6	23.0	16.4
Moran's I	8.8	9.1	10.3	9.2	7.1	7.2	7.9	8.9
SUV_{mean}	7.3	8.0	8.4	8.8	8.3	9.4	8.5	9.7
RMS	7.4	8.1	8.4	8.9	8.2	8.8	8.3	9.0
SUV_{peak}	8.6	9.1	10.1	9.2	8.6	9.1	10.1	9.1
Laplacian mean	9.0	10.0	12.2	10.4	10.9	12.8	11.3	13.1
SUV_{max}	10.0	8.3	10.3	10.0	10.0	8.3	10.2	10.0
TLG	12.4	9.6	10.9	9.4	10.0	8.7	10.0	8.9
SD	12.1	10.8	11.8	10.3	10.3	8.5	10.3	9.0
Mean absolute deviation	12.5	10.2	12.2	9.9	10.4	8.1	10.5	8.5
Median absolute deviation	12.5	10.2	12.3	9.6	10.4	8.2	10.5	8.8
Uniformity (FB)	11.6	9.2	12.3	8.3	12.6	8.5	13.4	11.7
SUV_{star}	14.0	12.3	12.1	10.3	13.5	12.4	11.8	10.5
SUV_{range}	13.4	10.1	12.2	12.0	12.5	10.5	12.6	11.0
Total energy	18.3	17.0	17.5	16.4	17.4	16.8	17.6	17.1
Variance	23.9	20.9	23.2	19.8	20.4	16.8	20.5	17.7
Skewness	21.6	19.2	33.9	84.2	55.6	84.9	44.7	60.5

*Two types of SUV discretization were applied: 64 grey level bins (64B) or a fixed bin size of 0.25 g/mL (FB). The features are sorted by the lowest TRT_a obtained for any combination of reconstruction and delineation.

Supplemental Table 10. Mean absolute test-retest variability (TRT_a, %) and standard deviation (SD) of various shape-based and texture-based (fractal) features.

	PET-based delineation				CT-based delineation			
	EANM-compliant reconstruction		PSF-based reconstruction		EANM-compliant reconstruction		PSF-based reconstruction	
Radiomics features*	Mean	SD	Mean	SD	Mean	SD	Mean	SD
Abundance	2.2	2.2	2.2	1.5	1.4	1.2	1.4	1.2
Lacunarity	3.3	9.6	3.9	11.5	1.7	7.3	1.7	7.3
FD	4.1	7.5	4.1	7.5	1.9	4.2	1.9	4.2
Radius of an equivolumetric sphere	2.9	1.8	3.2	2.1	1.9	1.2	1.9	1.2
Compactness A	5.3	3.9	6.3	5.1	3.7	3.2	3.7	3.2
Sphericity	4.0	3.3	4.3	3.7	4.6	3.5	4.6	3.5
Disproportion	4.0	3.3	4.3	3.7	4.6	3.5	4.6	3.5
$S2V_{eq}$	4.0	3.3	4.3	3.7	4.6	3.5	4.6	3.5
S2V	4.3	4.5	5.6	4.9	4.7	3.4	4.7	3.4
MATV/AV	8.6	5.4	9.6	6.4	5.7	3.6	5.7	3.6
Surface area	6.6	5.3	6.1	6.3	5.9	5.3	5.9	5.3
Compactness B	11.8	9.9	12.9	10.9	13.7	10.4	13.7	10.4

* The features are sorted by the lowest TRT_a obtained for any combination of reconstruction and delineation.

Supplemental Table 11. Mean absolute test-retest variability (TRT_a, %) and standard deviation (SD) of various texture-based features (based on grey-level co-occurrence matrices).

	PET-based delineation				CT-based delineation			
	EANM-compliant reconstruction		PSF-based reconstruction		EANM-compliant reconstruction		PSF-based reconstruction	
Radiomics features*	Mean	SD	Mean	SD	Mean	SD	Mean	SD
IDMN (FB)	0.7	0.5	0.9	0.4	0.6	0.4	0.5	0.4
IDMN (64B)	0.8	0.5	0.9	0.5	0.6	0.4	0.5	0.4
IDN (64B)	0.8	0.5	1.0	0.6	0.8	0.6	0.7	0.5
IDN (FB)	0.7	0.5	1.0	0.6	0.8	0.5	0.7	0.5
IMC2 (64B)	1.7	1.7	1.8	2.1	1.2	1.1	0.9	0.6
Sum entropy (64B)	2.2	1.4	2.3	1.3	1.3	0.8	1.2	0.9
Difference entropy (64B)	1.9	1.4	2.0	1.3	1.5	1.1	1.5	0.9
Entropy (64B)	2.6	1.6	3.1	1.8	1.5	0.9	1.5	0.8
IMC1 (64B)	4.6	3.9	4.3	5.0	3.8	3.0	2.7	1.9
Entropy (FB)	4.6	3.9	3.7	2.9	3.6	3.2	3.0	2.5
Sum entropy (FB)	4.5	3.8	3.3	2.8	3.6	3.2	3.1	2.8
IMC2 (FB)	4.2	4.7	4.5	3.8	3.6	3.3	3.3	3.3
Difference entropy (FB)	5.0	4.3	3.7	3.4	4.1	4.0	3.5	3.3
Sum average (64B)	4.1	3.3	4.9	3.3	4.7	3.9	5.3	3.8
Dissimilarity (64B)	4.1	2.6	5.2	2.7	5.4	3.4	5.6	3.1
IMC1 (FB)	5.3	7.5	9.1	7.1	7.9	4.6	8.9	6.5
Homogeneity 1 (64B)	7.4	7.8	8.3	6.7	5.7	4.5	5.9	4.0
Inverse variance (64B)	6.1	5.6	6.9	4.2	9.1	7.6	9.8	9.0
Cluster tendency (64B)	7.4	7.6	6.7	4.9	8.6	4.6	9.1	4.9
Homogeneity 1 (FB)	7.4	5.6	6.8	6.4	7.8	8.1	8.0	8.2
Variance (64B)	7.3	4.7	8.2	4.7	8.6	5.7	9.5	5.4
Autocorrelation (64B)	7.5	5.5	8.5	5.9	8.7	6.4	9.8	5.7
Sum variance (64B)	7.6	5.2	8.5	5.2	9.1	6.2	10.1	5.6
Contrast (64B)	7.6	5.3	9.0	5.2	10.4	6.6	10.3	7.7
Homogeneity 2 (64B)	10.1	10.4	11.2	8.7	7.9	6.6	8.1	6.6
Homogeneity 2 (FB)	9.6	7.5	9.3	8.1	10.3	10.5	10.6	10.4
Sum average (FB)	9.7	8.9	9.6	9.1	11.0	10.3	10.4	9.9
Correlation (FB)	21.1	18.3	48.4	84.5	10.3	10.4	9.6	10.9
Correlation (64B)	23.9	19.2	57.6	102	10.5	11.3	9.8	11.2
Inverse variance (FB)	10.8	8.8	12.0	12.5	13.5	13.3	15.3	16.6
Dissimilarity (FB)	12.2	8.7	11.2	10.3	11.9	11.2	11.3	10.8
Cluster prominence (64B)	13.4	13.6	12.2	9.8	16.7	7.9	17.8	9.6
Maximum probability (FB)	14.8	14.7	16.8	10.4	13.2	7.5	12.3	8.5
Maximum probability (64B)	16.5	15.6	16.9	12.9	12.7	8.1	12.5	7.9
Energy (64B)	20.1	18.1	21.2	12.7	13.8	11.9	14.0	10.9
Energy (FB)	17.5	14.1	18.8	8.5	16.5	12.6	15.5	12.7
Variance (FB)	19.9	17.8	19.2	18.3	21.5	19.5	20.6	18.6
Autocorrelation (FB)	19.6	17.7	19.7	17.4	21.9	19.4	21.4	18.1
Contrast (FB)	22.7	16.7	21.2	19.4	21.4	20.2	20.4	19.7
Sum variance (FB)	21.1	19.2	20.6	19.4	23.2	20.9	22.4	19.5
Cluster tendency (FB)	23.1	20.0	21.5	19.1	23.6	18.7	23.5	17.3
Cluster shade (64B)	116	207	46.9	64.6	44.0	84.4	24.0	17.6
Cluster shade (FB)	70.7	65.1	116	288	51.0	57.9	40.6	33.4
Cluster prominence (FB)	42.7	37.8	41.4	35.5	43.5	35.5	43.9	33.7

*Two types of SUV discretization were applied: 64 grey level bins (64B) or a fixed bin size of 0.25 g/mL (FB). The features are sorted by the lowest TRT_a obtained for any combination of reconstruction and delineation.

Supplemental Table 12. Mean absolute test-retest variability (TRT_a, %) and standard deviation (SD) of various texture-based features (based on grey-level run-length matrices).

**Radiomics features***	**PET-based delineation**				**CT-based delineation**			
	**EANM-compliant reconstruction**		**PSF-based reconstruction**		**EANM-compliant reconstruction**		**PSF-based reconstruction**	
	**Mean**	**SD**	**Mean**	**SD**	**Mean**	**SD**	**Mean**	**SD**
SRE (64B)	3.6	2.8	4.1	3.6	4.0	2.9	3.6	3.0
SRE (FB)	4.1	3.3	4.8	4.2	4.0	3.2	4.1	3.2
LGLRE (FB)	4.5	3.9	5.0	3.6	6.1	4.1	5.8	4.0
LGLRE (64B)	4.7	4.2	4.9	3.8	6.2	3.9	5.7	3.3
RLN (64B)	8.3	5.2	10.0	8.1	7.6	5.6	6.4	4.8
SRLGLE (64B)	6.7	5.1	8.6	6.7	8.2	9.5	7.6	8.5
RLN (FB)	10.1	5.6	11.9	7.8	7.5	5.3	7.0	5.1
SRLGLE (FB)	7.2	5.0	8.1	6.8	7.9	8.2	7.9	8.3
RP (64B)	8.3	8.5	8.4	7.3	9.5	5.1	9.8	5.0
SRHGLE (64B)	9.8	4.7	10.2	7.4	10.7	5.7	11.4	4.9
LRHGLE (64B)	10.1	4.8	9.9	7.5	9.9	5.7	10.4	5.0
HGLRE (64B)	9.9	4.7	10.2	7.4	10.6	5.6	11.3	4.9
GLN (FB)	13.2	9.7	14.1	11.8	13.9	9.5	14.2	9.9
RP (FB)	14.2	10.0	15.1	10.3	13.6	11.2	14.6	11.3
GLN (64B)	14.3	11.7	15.0	12.5	14.3	10.1	14.3	9.8
LRHGLE (FB)	15.0	13.3	15.6	15.2	17.9	16.9	18.7	17.1
LRE (FB)	17.6	10.8	18.4	13.6	18.5	12.2	18.3	12.2
LRE (64B)	18.2	11.6	18.3	13.9	17.8	12.2	17.9	12.1
LRLGLE (64B)	20.3	13.6	19.8	15.1	19.3	13.1	19.3	13.0
LRLGLE (FB)	20.0	13.0	20.0	14.9	20.0	13.1	19.8	13.0
HGLRE (FB)	20.4	16.2	19.8	17.5	21.8	20.4	21.6	19.1
SRHGLE (FB)	22.5	17.0	20.7	18.0	22.5	21.1	22.2	19.4

*Two types of SUV discretization were applied: 64 grey level bins (64B) or a fixed bin size of 0.25 g/mL (FB). The features are sorted by the lowest TRT_a obtained for any combination of reconstruction and delineation.
